# Spatio-Temporal Monitoring of Benthic Anatoxin-a-Producing *Tychonema* sp. in the River Lech, Germany

**DOI:** 10.3390/toxins14050357

**Published:** 2022-05-20

**Authors:** Franziska Bauer, Michael Stix, Bernadett Bartha-Dima, Juergen Geist, Uta Raeder

**Affiliations:** 1 Aquatic Systems Biology Unit, Limnological Research Station Iffeldorf, Technical University of Munich, Hofmark 1-3, D-82393 Iffeldorf, Germany; michael.stix1@gmx.de (M.S.); geist@tum.de (J.G.); uta.raeder@tum.de (U.R.); 2 Bavarian Health and Food Safety Authority, Veterinärstraße 2, D-85764 Oberschleissheim, Germany; bernadett.bartha-dima@lgl.bayern.de

**Keywords:** cyanobacteria, toxic bloom, cyanotoxins, neurotoxins, macrophytes, habitat, reservoir, public health

## Abstract

Incidents with toxic benthic cyanobacteria blooms have been increasing recently. In 2019, several dogs were poisoned in the river Lech (Germany) by the benthic anatoxin-a-producing genus *Tychonema*. To characterize spatial and temporal distribution of potentially toxic *Tychonema* in this river, a systematic monitoring was carried out in 2020, focusing on the occurrence of the genus, its toxin production and habitat requirements. *Tychonema* and cyanobacterial community composition in benthic mats and pelagic samples were identified using a combined approach of microscopy and DNA sequencing of the 16S rRNA gene. In addition, anatoxin-a concentrations of selected samples were measured using the ELISA method. The habitat was characterized to assess the ecological requirements and growth conditions of *Tychonema*. *Tychonema* mats and anatoxin-a were detected at several sampling sites throughout the entire study period. Toxin concentrations increased with the progression of the vegetation period and with flow direction, reaching values between 0 and 220.5 µg/L. Community composition differed among pelagic and benthic samples, with life zone and substrate condition being the most important factors. The results of this study highlight the importance of monitoring and understanding the factors determining occurrence and toxin production of both pelagic and benthic cyanobacteria due to their relevance for the health of humans and aquatic ecosystems.

## 1. Introduction

Cyanobacteria are a diverse and phylogenetically ancient group of organisms. They colonize a wide variety of habitats and play an important role as primary producers of fresh waters. Many representatives of these phototrophic organisms can produce toxins [1,2,3,4]. These cyanotoxins cause serious problems in drinking water supplies, bathing waters and fisheries, especially when toxic cyanobacteria form harmful mass populations, so called “cyanobacterial blooms” [5,6,7].

In the aquatic environment, cyanobacteria colonize two different types of habitats [8,9]: (1) Pelagic cyanobacteria thrive in the open water zone and can form blooms that float on the lake surface. (2) Benthic cyanobacteria settle on various substrates of the water bottom or on macrophytes [10,11]. These benthic mats can also detach from the substrate and then float on the water surface.

To date, research has globally mostly focused on the pelagic cyanobacteria. Because of this, their community compositions as well as the factors governing their mass developments are well characterized. Such risk factors for pelagic blooms result from eutrophication. Furthermore, it has been shown that pelagic cyanobacteria are promoted by climate change and that harmful pelagic cyanobacteria blooms will likely increase in the future [12,13,14].

In contrast to their pelagic conspecifics, benthic cyanobacteria have received less attention, and knowledge has mainly been derived from specific case studies. As a result, the reasons and risk factors for the spatio-temporal distribution patterns, and specifically of mass occurrence of benthic cyanobacteria, have not yet been clarified. However, benthic cyanobacteria are gaining growing attention, as they have been responsible for increasing poisoning incidents in recent years [15,16,17,18,19]. The rise in incidents involving toxic benthic cyanobacteria in the recent past suggests that climate change is also responsible for the growing importance of this group [20].

Many forms of benthic cyanobacteria like *Microcoleus*, *Oscillatoria*, *Phormidium* or *Tychonema* can produce highly potent neurotoxins [16,19,21,22]. When cyanobacterial mats detach from the bottom of the water body and float on the surface as conglomerates, there is a high risk of poisoning due to the high concentration of toxins in such mats. Oral ingestion is particularly dangerous, so that especially small children and animals are at risk due to their behavior [15,18,23,24].

In Germany, the first case of fatal neurotoxicosis associated with toxic cyanobacteria was observed in 2017: a tychoplanktic anatoxin-a producing *Tychonema* population led to the poisoning of several dogs in Lake Tegel, Berlin [25]. Two years later, a mass occurrence of benthic cyanobacteria of the same genus caused the poisoning of three dogs in 2019 at Lake Mandichosee, a reservoir of the river Lech, a major river in Southern Germany [19].

Immediately after the incident, a random screening was carried out to assess the situation on site. First results indicated the occurrence of *Tychonema* upstream along the entire river [19]. In Germany, a systematic procedure for early detection of such critical situations has neither been developed nor established, yet. Current routine monitoring programs only examine open water samples and do not target benthic cyanobacteria.

Because of the incidents in reservoir Mandichosee in 2019, detailed investigations on the occurrence of *Tychonema* were carried out in this lake and in all upstream Lech reservoirs in 2020. Since this group of organisms poses a threat to human health, knowledge on its distribution and abundance is of great interest. Consequently, the objectives of this study were (1) an analysis of *Tychonema* occurrence at the Lech reservoirs during the vegetation period in 2020 based on microscopic and genetic identification, (2) the quantification of its anatoxin-a in selected samples based on ELISA, and (3) the characterization and distribution of the habitats of the potentially anatoxin-a producing cyanobacteria in the system.

We hypothesized (i) the presence of both *Tychonema* and anatoxin-a at several sampling sites along the river Lech, (ii) temporal distribution patterns along the course of the river, with particularly high occurrences downstream later in the growing season, and (iii) spreading, mainly influenced by the variables temperature and substrate.

Detailed knowledge of the temporal and spatial occurrence of potentially toxic *Tychonema* in the River Lech is an important basis for adapting monitoring strategies and for providing early warning in the future. Both are important tools for the protection of human health.

## 2. Results

### 2.1. Occurrence and Distribution of Tychonema in the River Lech

**Figure 1:**
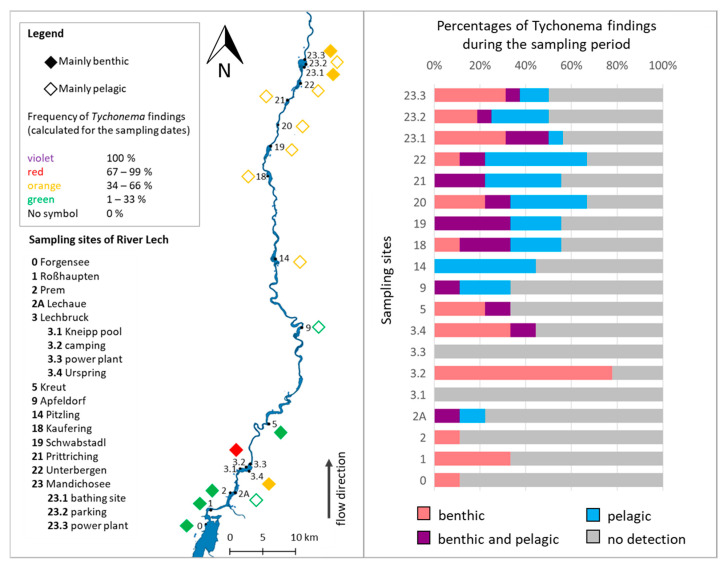
Frequency of positive PCR detections of *Tychonema* over the entire sampling period between May and October 2020. Data for this figure are based on the results of the PCR. (**Left**): Filled squares represent sampling sites with more than 50% benthic *Tychonema* findings, empty squares represent sampling sites with more than 50% pelagic *Tychonema* findings. (**Right**): More detailed presentation of *Tychonema* occurrence during the sampling period, showing all benthic and pelagic detections.

*Tychonema* was detected in 17 of the 19 sampling sites on at least one of the nine sampling days throughout the vegetation season. Samples were considered positive if there was a positive PCR detection of DNA from the pelagic and/or benthic organisms. No site had a positive *Tychonema* detection at every sampling event (Figure 1, right). At the sampling sites Lechbruck-Kneipp pool (3.1) and Lechbruck-power plant (3.3), no *Tychonema* occurrence was detected by PCR in 2020 (Figure 1). *Tychonema* was most frequently found during sampling at the sampling site Lechbruck-camping (3.2). There, *Tychonema* was found in 78% of the samples. However, only benthic *Tychonema* forms were identified, while the *Tychonema* detection in the pelagic samples was always negative. *Tychonema* was also frequently detected at the sampling site Lechbruck-Urspring (3.4) and at the more northerly sampling sites starting at Pitzling (14). At Pitzling, *Tychonema* was exclusively detected in the pelagic samples, but no benthic *Tychonema* occurrences were observed. At the other sampling sites, *Tychonema* was detected in both the benthic and pelagic samples, with detection prevailing in the pelagic samples. At reservoir Mandichosee, predominantly benthic *Tychonema* was detected at the sampling sites Mandichosee-bathing area (23.1) and Mandichosee-power plant (23.3), whereas mainly pelagic *Tychonema* was identified at the sampling site Mandichosee-parking (23.2).

### 2.2. Molecular Analyses

#### 2.2.1. Illumina MiSeq Sequencing

Sequencing of the 16S rRNA gene revealed 19 cyanobacterial genera in the benthic samples and 20 genera in the pelagic samples. Based on sequence data, the genus *Tychonema* was divided into two operational taxonomic units (OTUs) with 97% sequence identity corresponding to two species, one of them being only detected in small proportions at the sampling sites Lechbruck-camping (3.2) and at the reservoir Mandichosee (23). No difference to other *Tychonema* trichomes could be detected by microscopy.

In total, both benthic and pelagic cyanobacterial communities were examined in 54 samples. In 78% of these samples, *Tychonema* was identified in at least one of the two types of occurrences. In 50% of the *Tychonema*-positive samples, *Tychonema* was detected in both, benthic and pelagic communities. However, in 24% of the samples, *Tychonema* was exclusively detected in the benthic form only, whereas 26% were exclusively found as pelagic life forms. The proportions of *Tychonema* sequences in the benthic samples ranged from 0 to 100% at the site Apfeldorf (9; 5 May 2020), and from 0 to 79% in the pelagic samples at the site Prittriching (21; 26 May 2020) relative to the total cyanobacterial community.

**Figure 2:**
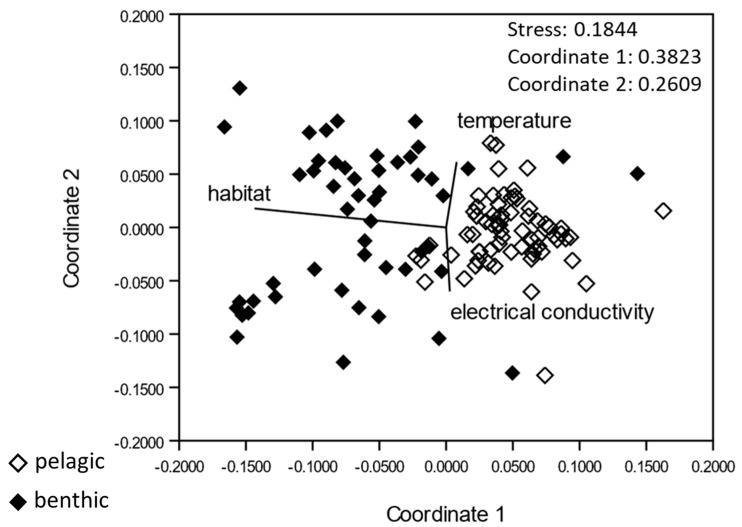
Non-metric multidimensional scaling of all the cyanobacteria sequences considering the environmental variables *habitat*, *temperature* and *electrical conductivity*. Unfilled rhombs represent pelagic samples, filled rhombs represent benthic samples and characterize the habitat of cyanobacteria.

The cyanobacterial communities of the benthic and pelagic habitats were very different as evident from the non-metric multidimensional scaling (NMDS). The NMDS identified “habitat” (i.e., benthic or pelagic) as the most important variable determining community composition. Less important were the variables “temperature” and “electrical conductivity” (Figure 2).

The cyanobacterial communities of the benthic samples were very diverse, comprising 19 cyanobacterial genera. Besides *Tychonema*, the genera *Leptolyngbya*, *Scytonema* and *Microcoleus* dominated (Figure S1). Other important genera were *Potamolimnea*, *Pseudanabaena*, *Calothrix* and *Geitlerinema*. In ten of the 51 benthic samples, *Tychonema* contributed more than 90%. Only at the sampling site Pitzling (14), no benthic *Tychonema* occurrence was detected. However, only one benthic sample was taken for sequencing from this site during the entire study period. The genera *Calenema* and *Wilmottia* were detected exclusively in the benthic samples.

The cyanobacterial communities of the pelagic samples were mostly dominated by the genus *Cyanobium* (Figure S2). In some pelagic samples from the sampling sites Lechaue (2A), Apfeldorf (9), Prittriching (21) and Mandichosee (sites bathing area, 23.1, and parking, 23.2) the genus *Tychonema* prevailed. In reservoir Mandichosee, the dominance of *Tychonema* in the pelagic cyanobacterial community was observed in autumn (October), at the other sampling sites at the beginning of the investigations in May. The following other cyanobacterial genera were frequently encountered in the pelagic samples: *Geitlerinema*, *Snowella* and *Pseudanabaena*. The genera *Limnothrix*, *Microcystis* and the species *Candidatus obscuribacter* were detected exclusively in the pelagic samples. In the mixed sample collected from reservoir Mandichosee at its deepest point on 27th October 2020, the genera *Geitlerinema*, *Tychonema*, *Kamptonema* and the species *Candidatus obscuribacter* were detected in addition to the main genus *Cyanobium*. At the time of this sampling, reservoir Mandichosee was almost completely mixed due to the onset of autumn circulation (please see further information on hydro-physical parameters in Section 2.5.1).

#### 2.2.2. Detection of Toxin Genes

Genes for the biosynthesis of the cyanotoxins anatoxin-a, microcystin and saxitoxin were not detected by PCR in any of the pelagic and benthic samples taken in the river Lech in 2020.

### 2.3. Macroscopic and Microscopic Appearance of Tychonema

**Figure 3:**
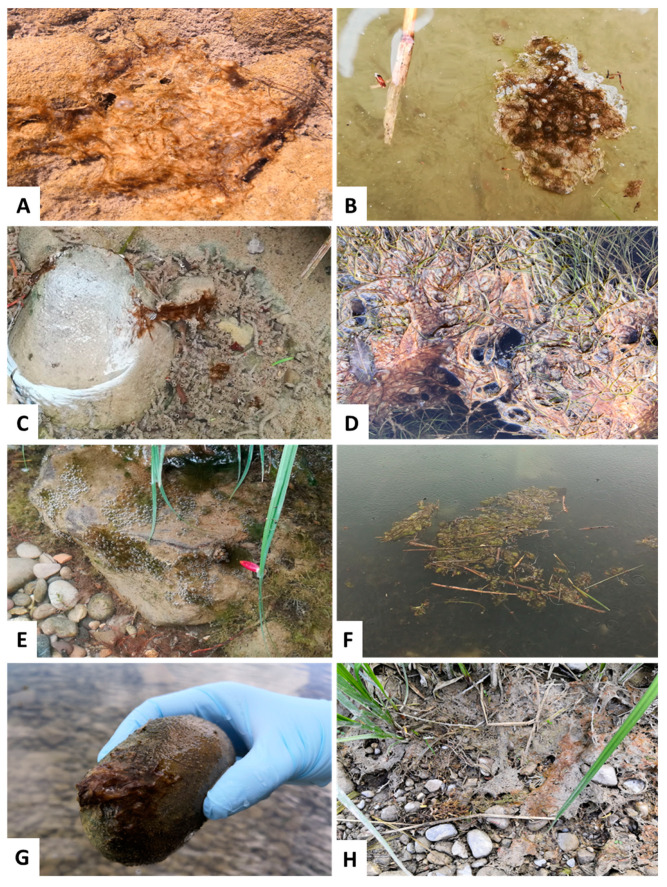
Macroscopic appearance of *Tychonema* sp. in the river Lech. (**A**) Fine *Tychonema* mat on stony sediment (very characteristic). (**B**) Small part of a floating *Tychonema* mat (very characteristic). (**C**) Reddish-brown *Tychonema* biofilm between stone and sediment. (**D**) Reddish-brown *Tychonema* biofilm on filamentous macrophytes. (**E**) Greenish *Tychonema* biofilm on a stone, the typical oxygen bubbles, produced by photosynthesis are visible. (**F**) Floating *Tychonema* mat associated with filamentous algae. (**G**) Reddish-brown *Tychonema* biofilm on a stone, slimy consistency very visible. (**H**) Dried *Tychonema* biofilm in the riparian area.

*Tychonema* often occurred as benthic biofilm on sediment, forming thin bacterial mats (Figure 3A). Foremost, such benthic mats were found on larger stones or gravelly sediment. However, *Tychonema* has also been detected in floating mats (Figure 3B). These *Tychonema* mats occurred in a wide range of sizes. Furthermore, *Tychonema* was found growing on macrophytes, whereby only washed-up dead plant parts could be examined during the study. The color of the *Tychonema* mats varied between reddish brown to purple, sometimes the mats appeared greenish. The consistency of *Tychonema* aggregates was slimy, and the filaments were often difficult to detach from each other and from the substrate.

**Figure 4:**
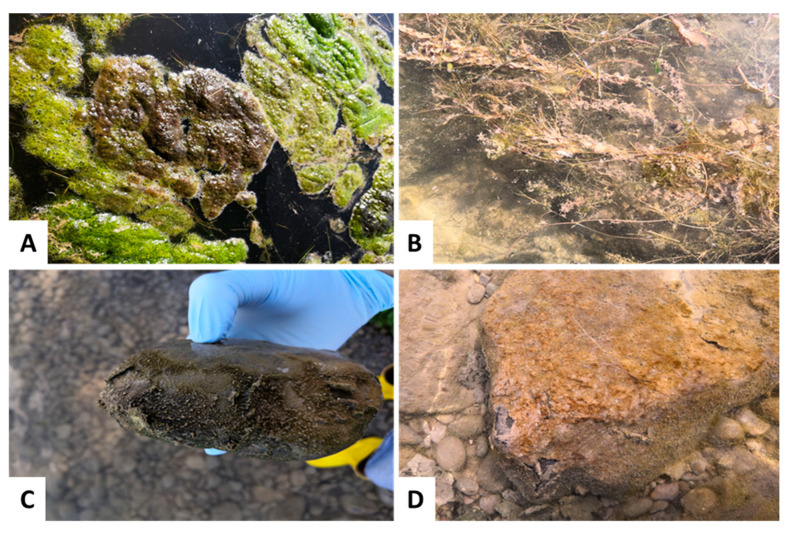
Appearances of other cyanobacteria and algae that could be easily misidentified as *Tychonema* based on their macroscopic characteristics. (**A**) Biofilm on filamentous algae. (**B**) Biofilm on macrophytes. (**C**) Greenish-brown crust on a stone. (**D**) Brownish biofilm on a stone.

*Tychonema* was not clearly identifiable based only on macroscopic morphology. There were other benthic algae and cyanobacteria that showed a similar appearance in the field (Figure 4), but where no *Tychonema* could be detected based on molecular genetic methods (please see further information on results of molecular genetic analyses in Section 2.2.1).

**Figure 5:**
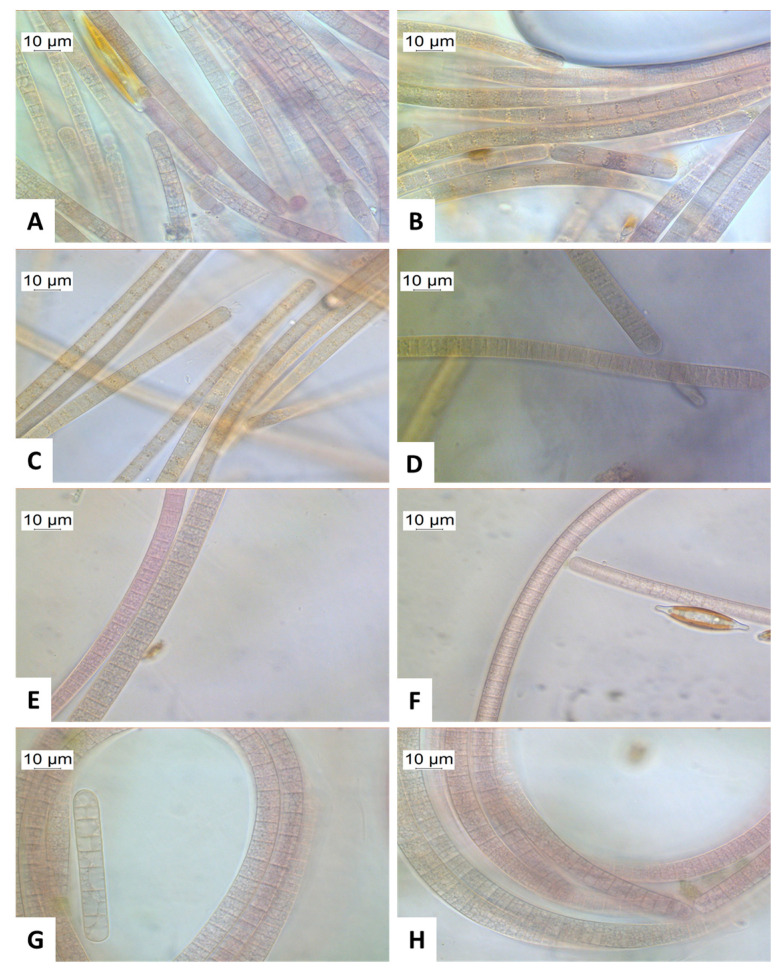
(**A**–**H**) Microscopic appearance of the *Tychonema* trichomes found in the river Lech in 2020 (1000× magnification).

All the benthic samples were analyzed microscopically at 1000× magnification. In the microscopic image, the color of the *Tychonema* cells often appeared light, olive green, grayish brown, or reddish brown to purple (Figure 5). The trichomes often reached lengths of several hundred micrometers. The individual cells of the trichomes were characteristically longer than wide. The cell walls were not always clearly visible. *Tychonema* cells do not have gas vesicles. However, distinct granules were often visible within the cells (Figure 5B) [26].

In addition to *Tychonema*, other filamentous cyanobacteria were also detected in the benthic samples. These were assigned to the order *Oscillatoriales* by microscopic analysis. The sequencing (please see further information on sequencing results in Section 2.2.1) showed that in addition to *Tychonema*, the *Oscillatoriales Microcoleus*, *Geitlerinema* and *Wilmottia* were present in the benthic samples.

### 2.4. Toxin Analyses

The anatoxin-a values of the analyzed samples ranged from 0.1 to 220 µg/L, with 56% of the samples having a value below 1.0 µg/L (Table 1).

The highest anatoxin-a concentrations with values above 100 µg/L were only measured towards the end of the vegetation period from September onwards. Furthermore, the high values were only detected downstream in the northern Lech reservoirs Schwabstadl (19) and Mandichosee (23). There was a general trend of increasing anatoxin-a values with the progression of the vegetation period and with the direction of flow from south to north.

### 2.5. Habitat Characterization

#### 2.5.1. Hydro-Physical Parameters

**Figure 6:**
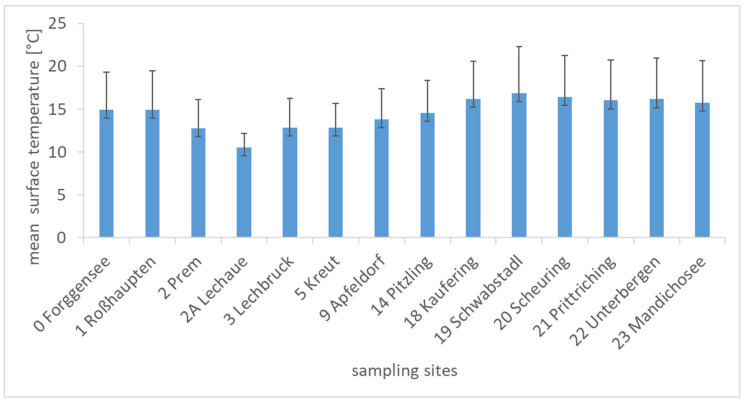
Mean surface temperatures and standard deviations at the sampling sites averaged over the study period. The numbers of the sampling sites correspond to the numbers of the reservoirs.

The average water surface temperatures at the different sampling sites ranged between 10.5 °C (Lechaue, 2A) and 16.9 °C (Schwabstadl, 19) over the sampling period (Figure 6). At the sampling sites between Lechaue (2A) and Schwabstadl (19), the average surface temperature increased continuously, reflecting the warming of the water body downstream. The average water temperature of the river Lech across all sampling sites and dates was 12.5 °C. *Tychonema* was detected in a temperature range between 6.5 and 23.5 °C. Due to the broad temperature distribution, no temperature preferences could be determined. A detailed presentation of all the hydro-physical parameters is shown in the supplementary (Table S1).

All sampling sites were aerobic with oxygen saturations between 80% and 149%. Particularly high values were measured at the following sampling sites: reservoir Mandichosee-power plant (23.3), reservoir Mandichosee-bathing site (23.1), Scheuring (20), Schwabstadl (19), Kaufering (18) and Mandichosee-parking (23.2).

The mean pH values of the different sampling sites were similar, varying between 8.3 and 8.9. Mean electrical conductivities varied between 315 and 520 µS/cm. The highest values were reached at Lechbruck-Kneipp pool (3.1) and Lechaue (2A). The averaged mean electrical conductivity tended to increase from South to North in the flow direction of the Lech River. Mean electrical conductivities at the sampling sites of reservoir Mandichosee ranged from 347 to 349 µS/cm.

The sampling at the deepest point of reservoir Mandichosee on 27 October 2020 took place with the onset of the autumn circulation. At this time, the lake was already almost completely mixed and had a temperature of 10.2 °C from the surface down to 4 m depth. Oxygen saturation was about 97%, pH was 8.3, and electrical conductivity was 380 µS/cm. Secchi depth was determined to be 2.5 m, corresponding to a euphotic zone of 6.3 m. Therefore, a major part of reservoir Mandichosee was euphotic and illuminated until the bottom of the lake, as a maximum water depth of 7.9 m was determined.

The flow velocity was below the measurement limit of 10 cm/s at all bank sampling sites, with one exception at the sampling site Lechbruck-Urspring (3.4). There, 15 cm/s were measured once, but this slight increase in flow velocity could be attributed to the wind.

#### 2.5.2. Substrate

We found various inorganic substrates at the study locations. At the sampling sites Prem (2) and Lechbruck-power plant (3.3), the substrate was formed by the concrete of the dam walls. This was classified as fine-grained matrix (clay). Fine material of the silt fraction was found in the Lechaue (2A), Lechbruck-Kneipp pool (3.1) and in Pitzling (14). A gravelly substrate dominated at all other sample sites.

We also found the following organic substrates. Macrophytes of the following genera grew or floated at the sampling sites: *Elodea*, *Hippuris*, *Myriophyllum*, *Potamogeton*, *Ranunculus* and *Schoenoplectus*. *Fontinalis* was found at the sampling site Kreut (5). In addition, the presence of filamentous algae was documented at many sampling sites. These filamentous algae often grew in association with *Tychonema*.

## 3. Discussion

The findings of this study show that benthic toxic cyanobacteria occurrences play an important role in the Lech river system, particularly since they have already led to several fatal poisonings of dogs. Based on the results of the spatio-temporal monitoring during 2020, it was shown that *Tychonema* was recurrent throughout the period from early spring to late fall across the entire river. The hypothesis that a temporal distribution pattern appears with mass occurrence downstream in late summer and autumn was not confirmed, although there tended to be an increase in detection during this period.

Furthermore, the hypothesis that temperature plays a major role in dispersal was not confirmed either. Nevertheless, toxin levels were found to increase as the growing season progressed and with increasing distance downstream.

*Tychonema* was found throughout the study area in the river Lech during the entire sampling period between May and October 2020. Besides *Tychonema*, the benthic samples were dominated by the genera *Leptolyngbya* (Synechococcales), *Scytonema* (Nostocales) and *Microcoleus* (Oscillatoriales). Furthermore, *Calothrix* (Nostocales) and *Geitlerinema* (Oscillatoriales) were detected multiple times. Genera detected exclusively in the benthic samples were *Wilmottia* (Oscillatoriales) and *Calenema* (Synechococcales). All these genera are filamentous cyanobacteria. Microscopically, they are often difficult to distinguish from *Tychonema*, stressing the importance of genetic confirmation as done in this study. *Tychonema* and *Microcoleus* are also phylogenetically very closely related [27]. *Microcoleus* is also known to produce anatoxin-a and dihydroanatoxin-a [22,28].

The pelagic samples were dominated by *Cyanobium.* This cyanobacterial genus is very common in the pelagial of many waters. Little is known about the toxicity of *Cyanobium*, but it has been recently shown that there are strains of this genus, that can produce Cylindrospermopsin [29]. Furthermore, besides *Tychonema*, the genus *Geitlerinema* was frequently detected and was also very dominant in the benthic samples. *Geitlerinema* was originally a subgenus of the genus *Phormidium* but is now a distinct genus within the order Oscillatoriales [30]. Some strains of *Geitlerinema* have been shown to produce a previously unknown cyanotoxin. These strains were originally isolated from a Brazilian reservoir [31]. The genus *Wilmottia,* which was detected in the benthic samples at the river Lech, is considered a new genus separated from the genus *Phormidium* [32]. Previously, *Wilmottia* was thought to occur only in cold and temperate regions of the world. However, it has now been proven that representatives of this genus can colonize a wide range of habitats [32,33]. The situation is similar with *Tychonema*, which is now also regularly encountered in Germany and Italy [19,25,34,35] and should thus no longer be considered a cold-stenothermic genus [36]. The genera *Scytonema*, *Microcoleus* and *Calothrix* can fix nitrogen [37], giving them a competitive advantage when nitrogen is scarce in the aquatic environment. In addition to *Tychonema*, *Microcoleus* may also be a producer of anatoxin-a in the river Lech. Besides, there are *Phormidium* strains that can produce anatoxin-a. Although the genus *Phormidium* was not detected during the study period, some representatives of new genera that have evolved from the genus *Phormidium* were identified. Therefore, it would be interesting in the future to investigate whether the anatoxins in river Lech originate only from *Tychonema* or are possibly also produced by other benthic genera. One problem occurred during the study: It was not possible to detect genes for the biosynthesis of anatoxin-a in the samples, although the toxin was present in many samples. We want to note that both methods are based on different principles. It is possible that not everything that is present on gene level is also present as a protein. Conversely, it is also possible that proteins are present without the genes being detected by the PCR, e.g., due to mutations in the primer binding regions. Therefore, we think that a combination of both methods provides the most reliable results. In any case, the presence of the toxin and therefore the detection of anatoxin by ELISA is clearly the evidence of higher toxicological relevance.

Anatoxin-a is a neurotoxin that inhibits the nicotinic receptor [38]. Acute toxicity of this cyanotoxin is characterized by rapid onset of paralysis, tremors, convulsions and finally death [38]. In 2019, poisonings with anatoxins led to the deaths of three dogs (Husky, Jack Russel Terrier and Yorkshire Terrier) in reservoir Mandichosee [19]. Anatoxin-a was also detected in selected samples during the season 2020. The highest concentrations of 220 µg/L were found in reservoir Mandichosee in autumn. A value above 100 µg/L was also determined once in Schwabstadl (19) in autumn. The maximum value of 220 µg/L was measured in a sample taken on 27 October 2020 next to the bathing area at reservoir Mandichosee. The guideline value for anatoxins in bathing waters was published in 2021 by the WHO and is 60 µg/L [39]. The median lethal dose (LD_50_) for oral ingestion is given between 10 and 25 mg/kg body weight [28]. In the 2019 study period, anatoxin-a concentrations in the samples reached values as high as 453 µg/L. The 2020 values were slightly lower in comparison but were in a similar range. In 2020, the highest values were again reached at the sampling site at the bathing area of reservoir Mandichosee [19]. When discussing anatoxin concentrations the limitation of the Abraxis anatoxin-a ELISA kit has to be kept in mind. The kit detects (+)anatoxin-a and homoanatoxin-a with an accuracy of 100.0% and 124.8%. However, it detects dihydroxyanatoxin-a and (−)anatoxin-a only with an accuracy of 1.0% and 0.3%. Due to the extremely low cross-reactivity with these two congeners, the anatoxin levels measured in the study may still be underestimated, as dihydroxyanatoxin-a and (−)anatoxin-a are hardly detected by the ELISA method.

Although the values were similar in both study years, it should be noted that—in contrast to 2019—no mass aggregation of *Tychonema* was observed in reservoir Mandichosee during the 2020 study year. This may be explained by differences in weather conditions published annually by the German Weather Service. For example, 2019 and 2020 were the third and second warmest years since weather records began in 1881. Average temperatures in Bavaria were 9.5 °C in both years. However, 2019 was characterized by more extreme fluctuations in terms of cold and hot spells. There were also significantly more hours of sunshine in 2019 (1885 h compared to 1595 h in 2020). 2019 was also characterized by heavy snowfall in the Alps and several very heavy rainfall events, all affecting the flow of the river Lech. These variable extreme weather events may have promoted the mass occurrence of *Tychonema* in 2019 and the associated toxin production. Thus, it is known that stress, in particular, promotes the production of toxic cyanobacteria [40].

Due to the missing mass aggregations, it would have been unlikely in 2020 for vulnerable individuals or even dogs to take up lethal doses of anatoxin-a by ingestion during the growing season. However, utmost caution should always be exercised in the future in the event of a renewed mass accumulation of *Tychonema*. For this purpose, it would be useful to educate the public not only to recognize pelagic cyanobacteria blooms, but also to be aware of the existence of potential toxic mats. Even though it has been shown that *Tychonema* cannot be identified by macroscopic features alone (please see further information on microscopy results in Section 2.3), benthic mats in general can certainly be recognized by laymen, at least in the shore areas (Figure 3). Based on the results of the 2019 and 2020 studies, it can be assumed that in the event of *Tychonema* mass assemblages in the algal mats, large amounts of anatoxin-a can be expected. If the anatoxin-a content per gram of fresh weight remains similar, the anatoxin-a content in the water body is likely to increase proportional to the *Tychonema* biomass. One study found that high temperatures or high UVB radiation can lead to rapid degradation of anatoxin-a [41]. However, degradation produces metabolites such as dihydroanatoxin-a [42], the toxicity of which has been the subject of studies with conflicting conclusions. Previous studies indicate that dihydroanatoxin-a is about ten times less toxic than anatoxin-a [43,44]. In contrast, a recent study revealed that oral ingestion of dihydroanatoxin-a is significantly more toxic than ingestion of anatoxin-a [28]. In the samples of the 2019 studies, in addition to anatoxin-a, dihydroanatoxin-a was detected in high concentrations by LC-MS/MS [19]. The samples of the vegetation period 2020 were only analyzed with regard to the anatoxin-a content.

Combining the findings of the PCR detection with the sequencing data, *Tychonema* was found at every sampling site on at least one sampling date. Less frequently, *Tychonema* was detected at the southern sampling sites, where the benthic forms of the genera dominated. Towards the north, *Tychonema* was detected more frequently. At sampling sites from Pitzling (14) to Unterbergen (22) and at Reservoir Mandichosee parking area (23.2), *Tychonema* was detected primarily in the open water. Since there are also sampling sites where *Tychonema* was detected exclusively in the benthic samples, it can be assumed that the filaments grew very locally in the mat. Therefore, no detection occurred in open water. Thus, the *Tychonema* cells that were detected in open water are most likely a pelagic form. Initially, it was considered that this might be a different species. However, based on the sequence data, this assumption must be rejected, as both forms belong to the same *Tychonema* OTUwith 97% sequence identity (species). Thus, it is more likely that hormogonia were detected in the open water samples. Hormogonia consist of specialized cells, that are found in many filamentous cyanobacteria genera, and serve reproductive purposes. These hormogonia are few-celled filamentous fragments that are often capable of creeping movements and thus serve to disperse cyanobacteria [37,45]. It has been observed in studies that hormogonia are also often formed when cultures that are in stationary phase are transferred to a new medium [45]. In the genus *Tychonema*, the trichomes dissolve into multiple hormogonia that are mostly immobile but sometimes capable of creeping movements [26].

At a large area of the sampling sites, gravelly sediment dominated, which apparently represents a suitable substrate for *Tychonema*. *Tychonema* assemblages were also often detected on particularly large stones. The sampling sites dominated by fine material of the silt fraction were Lechaue (2A), Lechbruck-Kneipp pool (3.1) and Pitzling (14). No benthic *Tychonema* assemblages were detected at Pitzling (14) at any time, and no benthic or pelagic *Tychonema* were detected at Lechbruck-Kneipp pool (3.1). These results indicate that the substrate may play an important role in the occurrence of *Tychonema*. This can be explained (1) by the hydrophobic nature of the cells that favors adhesion on the substrate and (2) by the increased light attenuation in fine sediment.

The adhesion mechanism of many benthic cyanobacteria relates to the surface of the filaments having hydrophobic properties. Thus, hydrophobicity appears to be the most important factor enabling bacterial adhesion to the substrate [46]. Hydrophobic exopolysaccharides are produced that enable or support anchorage to sediment or even plant material [47]. It was shown in studies that all benthic cyanobacteria examined were hydrophobic, whereas all pelagic cyanobacteria examined, including hormogonia, exhibited hydrophilic properties [46,47]. Another reason why *Tychonema* may prefer gravelly substrate is probably that light attenuation is higher in fine sediment. Because this sediment is much more flexible and lacks stability, light attenuation is greater than on gravelly sediment [48]. The fine substrate is a dynamic substrate and particles, or cell debris more often cover the benthic organisms on this substrate [48]. No temperature preferences were found in the study for the occurrence of *Tychonema*. Nevertheless, toxin levels were shown to be higher in fall and in the study sites with higher mean temperatures. Apparently, the temperature has less of an effect on the spread and more on the toxicity.

## 4. Conclusions

This study highlights the need for further research to fill the knowledge gaps in the field of benthic cyanobacteria. This hitherto largely overlooked group of cyanobacteria is certainly present not only in the river Lech, but also in many other rivers and in lakes or reservoirs, where it colonizes not only the riparian zones, but also deeper sediment layers that are not visible from the bank line. It can therefore be assumed that benthic cyanobacteria can grow over a very large area in clear water bodies. As soon as these mats detach and float to the water surface, they pose a great danger to small children and dogs. The fact that the ecological requirements of *Tychonema* have been poorly studied so far, emphasizes the need for culturing experiments to identify optimal growth conditions for this genus. Furthermore, the monitoring and precise analysis of benthic cyanobacteria communities including their toxigenic and toxic potential is of great importance. Currently, the mass occurrences of benthic cyanobacteria are not predictable, making early warning difficult. Climate change may play an important role in the increasing problem with toxic benthic cyanobacteria. Heavy rainfall and flooding lead to increased nutrient inputs to water bodies, favoring cyanobacteria. Prolonged periods of heat and drought lead to a warming of all water bodies and a reduction in flow velocity in streams, so that benthic algal mats can establish.

## 5. Materials and Methods

### 5.1. Study Site

The investigations were carried out in the German part of the river Lech between Forggensee in the south and reservoir Mandichosee in the north. The river Lech, which runs from south to north, is one of the largest and most important rivers in Bavaria, Southern Germany. The reservoir Mandichosee is a popular water, which is used intensively for leisure activities and recreation. For more detailed information on the river Lech and reservoir Mandichosee, see also [19].

### 5.2. Sampling and Post-Processing of the Samples

**Figure 7:**
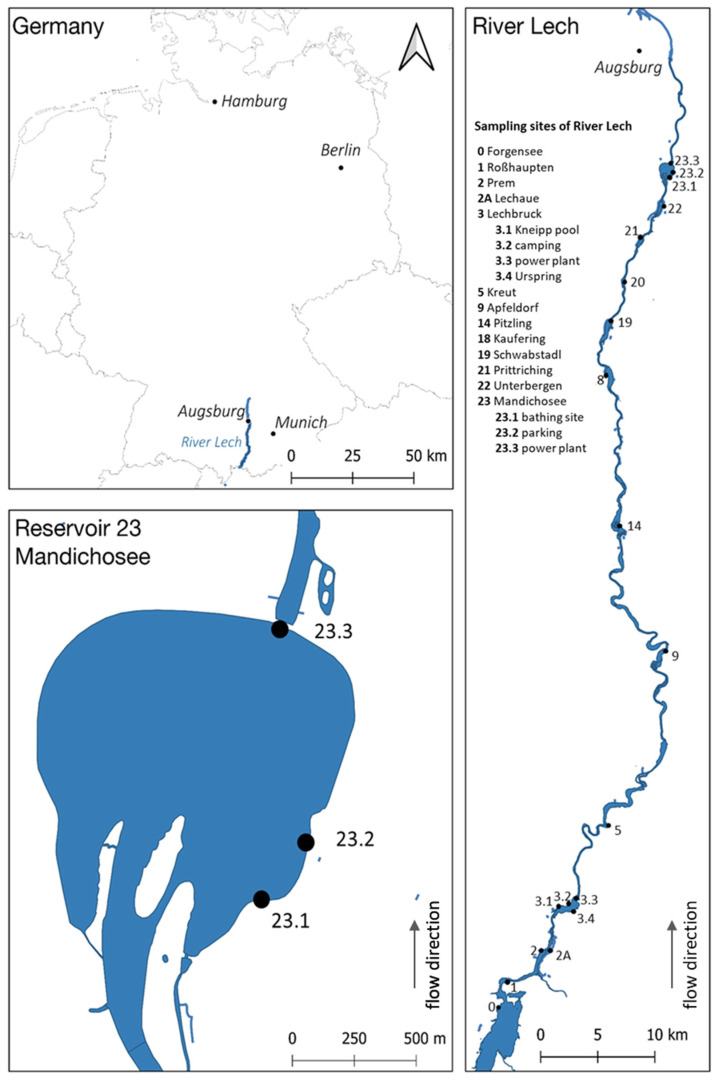
Map of the study area (according to [19], slightly modified). Sampling sites (dots) in the river Lech and reservoir Mandichosee. The numbers of the dams correspond to those of the public environmental agency.

The map (Figure 7) shows the selected sampling sites that had already been screened in the preliminary study in 2019 [19]. The sampling was conducted between 21 April 2020 and 23 November 2020. Until 26 May 2020, all sampling sites along the river Lech were investigated biweekly with the intention of recording the first occurrence of *Tychonema* at the selected sites as precisely as possible. Subsequently, sampling sites 0–22 were sampled in monthly intervals. The reservoir Mandichosee (dam 23) was continuously sampled biweekly. Sampling during monitoring was always conducted close to the bank, approximately 2 m away from bank line. In addition, a single sampling was conducted in the middle of reservoir Mandichosee on 27 October 2020, where a mixed sample was collected from a boat.

During each sampling, one liter of water was collected for the pelagic organism study. The water was filtered through 0.2 µm filters (Sartorius, Goettingen, Germany) in the laboratory so that the cells were enriched on the filters. The filters were frozen at −20 °C until further processing. For the study of benthic organisms, biomass was collected and placed in 50 mL falcon tubes with some water. Two samples were taken from each benthic site, one for molecular genetic analyses and microscopy, another one for toxin analyses, which were carried out at the Bavarian Health and Food Safety Authority, Oberschleissheim, Germany. The samples in the falcon tubes were frozen at −20 °C until further processing.

### 5.3. Molecular Detection

#### 5.3.1. DNA Extraction and Purification

DNA was extracted from the filters or the Eppendorf tubes using a phenol/chloroform-based method described by Zwirglmaier et al. [49]. Subsequently DNA was purified using Sure Clean Plus (Bioline, London, UK) and eluted in sterile ultrapure water. The DNA was then stored frozen at −80 °C until further analysis.

#### 5.3.2. Polymerase Chain Reaction

The polymerase chain reaction was performed to detect the target genes for anatoxin-A (ATX), microcystin (MCY) and saxitoxin (SXT) and *Tychonema*. Primer pairs for the target genes *anaC*, *mcyE* and *sxtA* were anxgen-F and anxgen-R [50], mcyE-F2 and mcyE-R4 [51] and sxtA-F and sxtA R [52]. *Tychonema* sp. were detected using the primer pair rbcLX-tycF and rbcLX-tycR [53]. In-house analyses showed that this primer pair does not only detect *T. bourrellyi*, but also *T. bornetii*. The strain PCC6506 (*Oscillatoria* sp., obtained from the Pasteur Culture Collection of Cyanobacteria, Paris, France) served as positive control for *anxC*. The strain SAG14.85 (*Microcystis aeruginosa*, obtained from the Culture Collection of Algae at Göttingen University, Goettingen, Germany) served as positive control for *mcyE,* and strain NIVA-CYA655 (*Aphanizomenon gracile*, obtained from the Norwegian Culture Collection of Algae, Oslo, Norway) as positive control for *sxtA*. The strain NIVA-CYA96/3 (*Tychonema bourrellyi*, obtained from the Norwegian Culture Collection of Algae, Oslo, Norway) served as a positive control for *Tychonema* sp. The PCR analyses were conducted with a CFX thermocycler (CFX Connect, Biorad, Feldkirchen, Germany) using the following protocol: an initial denaturation at 94 °C for 10 min followed by 30 cycles of 94 °C for 30 s (denaturation), annealing for 30 s, 72 °C for 30 s (extension), followed by a final elongation step of 72 °C for 7 min. Annealing temperatures were 55.8 °C for *anaC*, 56 °C for *mcyE*, 57.5 °C for *sxtA* and 58.8 °C for the *rbcLX* gen *of Tychonema* sp. To exclude contamination, each PCR was carried out under observation of negative controls. These were negative in all PCR assays. To ensure that the correct fragment was amplified in each case, the amplicon lengths were checked by gel electrophoresis. To size fragment length on the agarose gels, the Mass Ruler DNA Ladder Mix (Thermo Fisher Scientific, Waltham, MA, USA) was used. Samples were considered positive for *Tychonema* if there was a positive PCR detection of DNA from the pelagic and/or benthic organisms.

#### 5.3.3. Sequencing

Samples were sequenced bidirectionally at the Core Facility Microbiome, ZIEL, TUM in Freising using Illumina MISeq v3 2 × 300 paired-end sequencing. Polymerase chain reaction (PCR) primers used for the first step were S-DBact-0341-b-S-17 (5′ *TCGTCGGCAGCGTCAGATGTGTATAAGAGACAG*CCTACGGGNGGCWGCAG 3′) and S-D-Bact-0785-a-A-21 (5′ *GTCTCGTGGGCTCGGAGATGTGTATAAGAGACAG*GACTACHVGGGTATCTAATCC 3′) (Illumina overhang adapter in italics). These primers cover the 16S rRNA gene variable regions V3–V4. These hypervariable regions combined with a paired-end sequence configuration are recommended as the most effective study design [54]. Illumina MiSeq sequence data were processed within IMNGS [55]. The operational taxonomic units (OTUs) were clustered at 97% sequence identity and subsequently classified with SINA online [56]. For classification, SILVA taxonomy [57] was used, which was implemented in SINA online. To create an ordination, we chose a non-metric multidimensional scaling based on the distance matrix of our sequencing data in combination with selected environmental variables. Since the dataset includes zero values, we chose a Bray-Curtis similarity matrix and performed an NMDS ordination, where it is possible to choose any similarity matrix [58]. Non-metric multidimensional scaling (NMDS) was calculated within the software package PAST [59]. Sequencing was carried out in three runs. Results were merged before analysis. Sequence data have been submitted to NCBI’s Sequence Reads Archive (BioProject ID PRJNA783538).

### 5.4. Morphological Examinations

#### 5.4.1. Macroscopy

During each sampling, it was recorded whether benthic algae that could visually be assigned to *Tychonema* were detected in the riparian area of the water bodies. Particular attention was paid to floating algal mats on the water surface, to macrophytes washed-up to the riparian line, and to biofilms on different substrates, e.g., on stones.

#### 5.4.2. Microscopy

Algal or cyanobacterial trichomes were isolated immediately after sampling using a sterile inoculating loop and transferred to slides. Samples were derived from benthic mats, from floating algal material, or from the biofilm growing on the macrophytes or other substrates. Subsequent microscopic analysis was performed using a microscope (Leica DMRBE, Leica, Wetzlar, Germany) equipped with a camera (Zelos 285 GV, Kappa, Norderstedt, Germany). Trichomes suspected to be *Tychonema* were morphologically characterized and photographed. In addition, benthic samples were fixed with Lugol’s solution, allowing further microscopic analysis later if needed.

### 5.5. Toxin Analyses

The anatoxin-a concentrations were determined by ELISA (enzyme-linked immunosorbent assay) in the samples in which *Tychonema* had already been confirmed microscopically. Measurements were performed using the anatoxin-a ELISA Plate Kit by Eurofins Abraxis (Eurofins, Warminster, PA, USA). The ELISA method was based on the principle of a direct competitive ELISA. The samples, usually 50 ml of water, sediment and biomass, were frozen and thawed three times in succession for cell lysis to release the intracellular anatoxin-a present in the cells. After this procedure, the samples were homogenized using a vortex mixer (Scientific Industries, Bohemia, New York, NY, USA). Subsequently, after sedimentation of the biomass fraction, 10 ml of the supernatant was collected for toxin analysis according to the manufacturer’s specification. The concentrations of the samples were determined using a standard curve generated for each run, including the measurement of blank samples. Samples with an anatoxin-a concentration of more than 10 µg/L were diluted fifty-fold and reanalyzed for more accurate determination of the toxin content, otherwise samples were measured once. Finally, a total of 39 samples were analyzed.

### 5.6. Habitat Specification

#### 5.6.1. In Situ Measurements of Hydro-Physical Variables

Directly during sampling, the physical variables temperature, pH, electrical conductivity and oxygen content or saturation were determined using a multiparameter probe (MPP 930 IDS, WTW, Weilheim, Germany). The measurements were taken at the water surface in the area close to the shore, approximately 2 m from the water’s edge. During the collection of the mixed sample at reservoir Mandichosee on 27 October 2020, a depth profile of the physical parameters was also obtained from the surface to a depth of 6 meters. This depth profile was taken from a boat in the middle of the lake. Measurements were taken in meter increments during this process. During this sampling, the visible depth was also determined using a Secchi disk. The euphotic zone, i.e., the zone flooded with light, is calculated by multiplying the Secchi depth by a factor of 2.5.

At each sampling site in the riparian area, the flow velocity was determined at least once during the monitoring. For this purpose, a diving stick according to Jens was used in line with the manufacturer’s specifications [60]. The lower measurement limit is 10 cm/s.

#### 5.6.2. Substrate Characterization

The inorganic substrate was classified by means of grain size distribution. The different fractionations are listed in Table 2.

Organic substrates were macrophytes and filamentous green algae. For each sampling, it was noted which macrophytes were found at the sampling sites. A distinction was made whether the macrophytes and filamentous green algae were growing on site, floating on the water surface, or washed-up.

## Figures and Tables

**Table 1:** Anatoxin-a concentrations measured in selected samples.

	Sampling Site	Sampling Date	[µg/L] ATX
0	Forggensee	15 September 2020	0.2
0	Forggensee	13 October 2020	0.6
1	Roßhaupten	23 June 2020	9.2
1	Roßhaupten	21 July 2020	9.1
1	Roßhaupten	15 September 2020	0.2
2A	Lechaue	22nd April 2020	8.7
2A	Lechaue	5 May 2020	0.6
3.2	Lechbruck-camping	15 September 2020	0.6
3.2	Lechbruck-camping	13 October 2020	4.6
3.2	Lechbruck-camping	10 November 2020	0.4
3.4	Lechbruck-Urspring	22 April 2020	0.2
3.4	Lechbruck-Urspring	5 May 2020	0.1
3.4	Lechbruck-Urspring	15 September 2020	0.1
5	Kreut	22 April 2020	0.4
5	Kreut	5 May 2020	0.5
9	Apfeldorf	5 May 2020	1.8
18	Kaufering	5 May 2020	0.2
18	Kaufering	15 September 2020	0.2
18	Kaufering	10 November 2020	15.7
19	Schwabstadl	5 May 2020	0.2
19	Schwabstadl	13 October 2020	40.9
19	Schwabstadl	10 November 2020	212.5
21	Prittriching	5 May 2020	1.1
21	Prittriching	21 July 2020	11.2
22	Unterbergen	21 July 2020	0.6
22	Unterbergen	15 November 2020	13.0
22	Unterbergen	13 October 2020	89.6
23.1	Mandichosee-bathing site	4 August 2020	0.2
23.1	Mandichosee-bathing site	1 September 2020	0.2
23.1	Mandichosee-bathing site	15 September 2020	218.4
23.1	Mandichosee WW	29 September 2020	0.1
23.1	Mandichosee-bathing site	13 October 2020	144.8
23.1	Mandichosee-bathing site	27 October 2020	220.5
23.1	Mandichosee-bathing site	10 November 2020	2.3
23.2	Mandichosee-parking	4 August 2020	0.2
23.2	Mandichosee-parking	1 September 2020	0.2
23.3	Mandichosee-power plant	1 September 2020	0.2
23.3	Mandichosee-power plant	27 October 2020	0.8
23.3	Mandichosee-power plant	10 November 2929	143.8

**Table 2:** Classification of sediment.

Sediment Group	Clay	Silt	Sand	Gravel	Stones	Blocks
Grain size [mm]	<0.0002–0.0002	0.0002–0.063	0.063–2	2–63	63–200	>200

## Data Availability

Not applicable.

## Supplementary Materials

The following are available online at https://www.mdpi.com/article/10.3390/toxins14050357/s1, Table S1: Hydrophysical parameters of all samples, Figure S1: Cyanobacterial community of the benthic samples based on Illumina MiSeq sequencing data, Figure S2: Cyanobacterial community of the pelagic samples based on Illumina MiSeq sequencing data.
