# Supplementary material for: Spatio-Temporal Monitoring of Benthic Anatoxin-a-Producing Tychonema sp. in the River Lech, Germany
Source: Toxins (Basel). 2022 May 20;14(5):357. doi: 10.3390/toxins14050357 (PMC9144322; doi:10.3390/toxins14050357)
Supplement: Supplementary file 1 [file toxins-14-00357-s001.zip › toxins-1732443-supplementary.pdf]

toxins

MDPI

Article

Spatio-Temporal Monitoring of Benthic Anatoxin-a-Producing *Tychonema* sp. in the River Lech, Germany

Franziska Bauer, Michael Stix, Bernadett Bartha-Dima, Juergen Geist and Uta Raeder

Table S1. Hydrophysical parameters of all samples and values of the anatoxin-a measurements.

	Sampling Site	Temperature [°C]	Oxigen Saturation [%]	pH	Electrical Conductivity [µS/cm]
**0**	Forggensee	8.2–20.9	99–114	8.3–8.8	290–339
**1**	Roßhaupten	8.4–21.2	99–120	8.3–8.8	290–345
**2**	Prem	8.3–17.6	100–107	8.3–8.9	297–342
**2A**	Lechaue	8.4–12.9	95–108	7.9–8.4	442–528
**3.1**	Lechbruck-Kneipp pool	6.7–19.4	80–200	8.0–8.6	451–597
**3.2**	Lechbruck-camping	7.0–16.4	82–114	8.1–8.6	376–473
**3.3**	Lechbruck-power plant	8.1–16.2	96–115	8.3–8.6	298–344
**3.4**	Lechruck-Urspring	7.0–15.9	99–125	8.3–8.6	310–450
**5**	Kreut	8.3–16.2	100–117	8.3–8.8	309–353
**9**	Apfeldorf	8.0–18.4	83–111	8.3–8.5	319–398
**14**	Pitzling	8.6–18.8	98–119	8.3–8.7	323–483
**18**	Kaufering	8.7–21.2	103–149	8.4–8.8	314–391
**19**	Schwabstadl	8.2–23.3	101–151	8.2–8.9	332–402
**20**	Scheuring	8.7–22.1	102–154	8.5–8.8	311–393
**21**	Prittriching	8.6–21.7	102–133	8.4–8.9	317–395
**22**	Unterbergen	8.9–22.4	103–140	8.4–8.8	316–396
**23.1**	Mandichosee-bathing site	6.8–23.5	95–162	8.3–8.8	312–400
**23.2**	Mandichosee-parking	6.8–23.0	94–147	8.3–8.7	312–400
**23.3**	Mandichosee-power plant	6.5–21.7	101–188	8.2–8.8	317–387

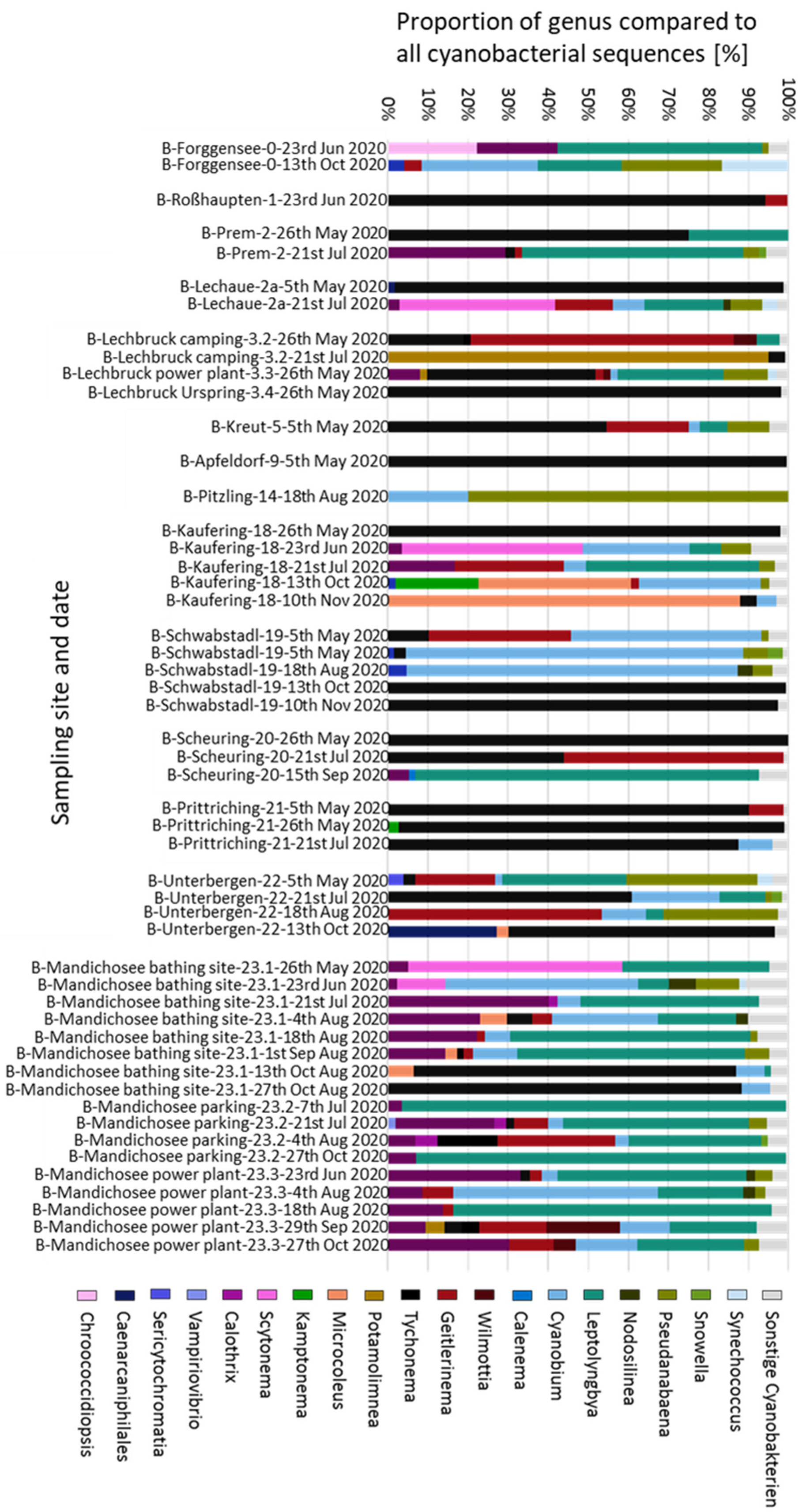

Figure S1. Cyanobacterial community of the benthic samples based on Illumina MiSeq sequencing data.

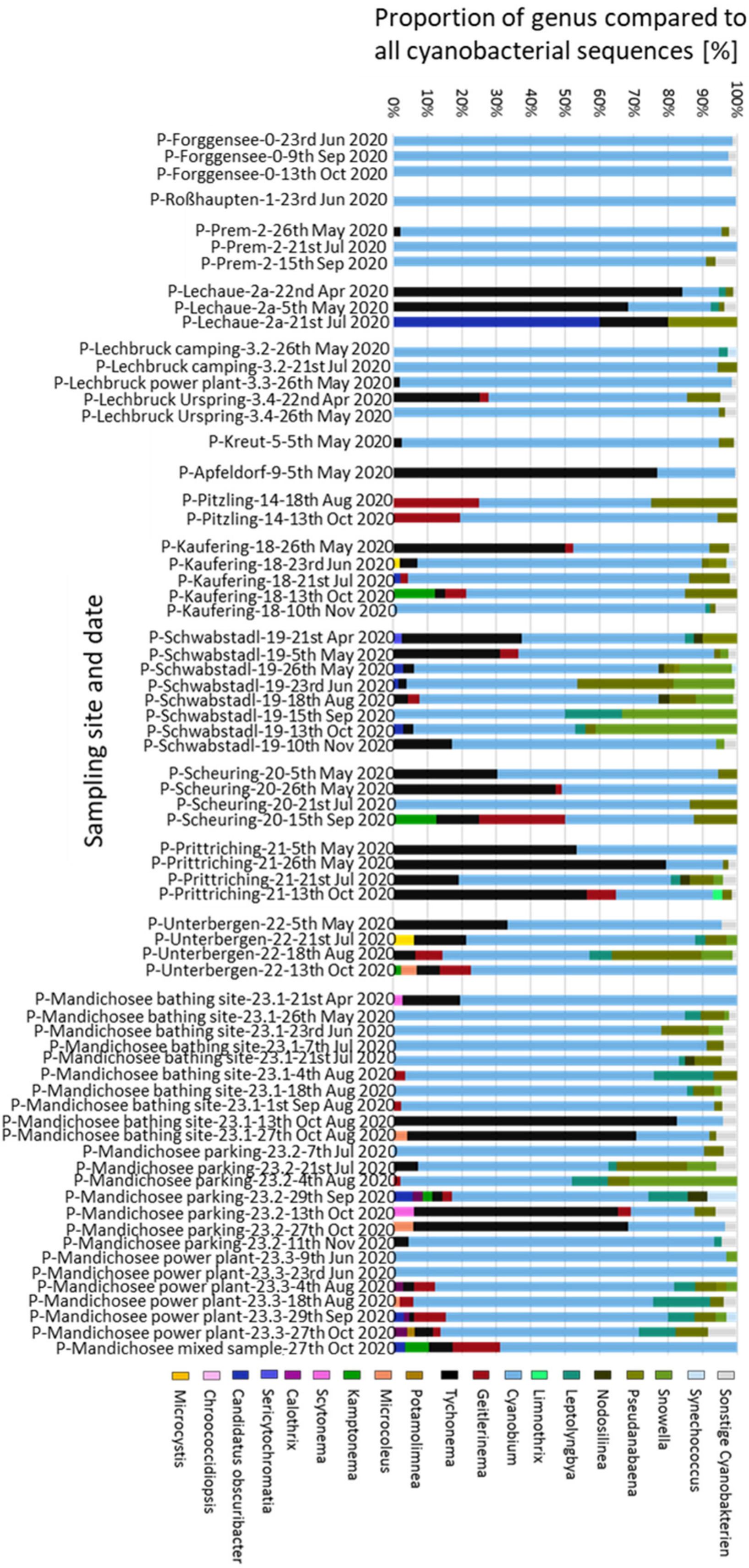

Figure S2. Cyanobacterial community of the pelagic samples based on Illumina MiSeq sequencing data.
